# Metabolomics in postmortem cerebrospinal fluid diagnostics: a state-of-the-art method to interpret central nervous system–related pathological processes

**DOI:** 10.1007/s00414-020-02462-2

**Published:** 2020-11-12

**Authors:** Simone Bohnert, Christoph Reinert, Stefanie Trella, Werner Schmitz, Benjamin Ondruschka, Michael Bohnert

**Affiliations:** 1 Institute of Forensic Medicine, University of Wuerzburg, Versbacher Str. 3, 97078 Wuerzburg, Germany; 2 Institute of Biochemistry and Molecular Biology I, Biozentrum - Am Hubland, 97074 Wuerzburg, Germany; 3 Institute of Legal Medicine, University Medical Center Hamburg-Eppendorf, Butenfeld 34, 22529 Hamburg, Germany

**Keywords:** CSF, Cerebrospinal fluid, Forensic neuropathology, Forensic neurotraumatology, Biomarker, Metabolomics

## Abstract

In the last few years, quantitative analysis of metabolites in body fluids using LC/MS has become an established method in laboratory medicine and toxicology. By preparing metabolite profiles in biological specimens, we are able to understand pathophysiological mechanisms at the biochemical and thus the functional level. An innovative investigative method, which has not yet been used widely in the forensic context, is to use the clinical application of metabolomics. In a metabolomic analysis of 41 samples of postmortem cerebrospinal fluid (CSF) samples divided into cohorts of four different causes of death, namely, cardiovascular fatalities, isoIated torso trauma, traumatic brain injury, and multi-organ failure, we were able to identify relevant differences in the metabolite profile between these individual groups. According to this preliminary assessment, we assume that information on biochemical processes is not gained by differences in the concentration of individual metabolites in CSF, but by a combination of differently distributed metabolites forming the perspective of a new generation of biomarkers for diagnosing (fatal) TBI and associated neuropathological changes in the CNS using CSF samples.

## Introduction

**Fig. 1:**
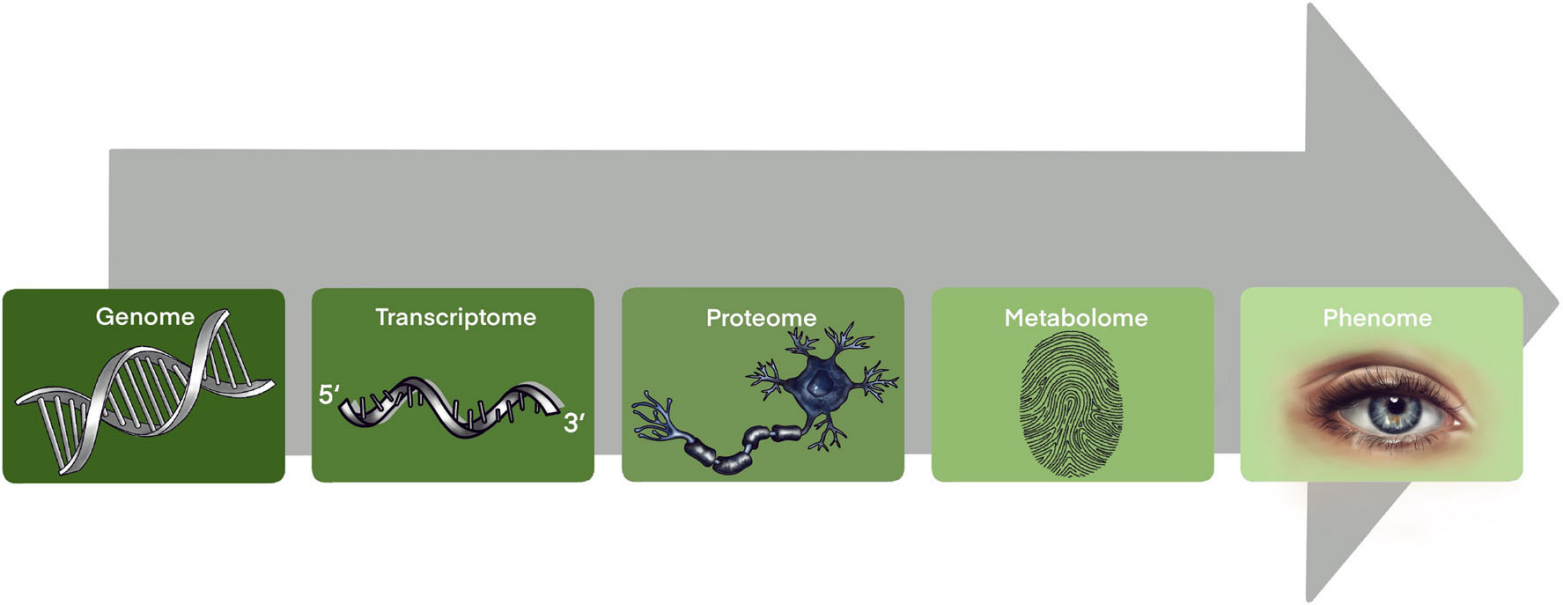
Biochemical flow diagram. The flow of biochemical information proceeds from the genome through the transcriptome and proteome and finally to the metabolome before presenting as the phenome (clinical manifestation)

In the last few years, quantitative analysis of metabolites in body fluids using LC/MS has become an established method in diagnostic medicine, which is applied both in routine diagnostic tests as well as in clinical studies. In the clinical laboratory, analysis of the metabolome is used especially for identifying biomarkers for predisposition, diagnosis, or treatment of various diseases. By investigating metabolic changes associated with a special disease, scientists hope to gain a better understanding of the pathophysiology and individual risk assessment of complex illnesses such as diabetes mellitus, coronary heart disease, or cancer [1–5]. Metabolites or “small molecules” are responsible for cellular signal transduction and energy balance; they reflect the biochemical status, i.e., the molecular fingerprint of an organism prior to its phenotypical presentation (see Fig. 1**)**.

With the help of metabolite profiles in biological specimens, physicians are able to understand pathophysiological mechanisms—such as the development of diseases or the traumatization of tissue [6]—at the biochemical and thus the functional level. An additional advantage of this approach is that the metabolites and metabolic pathways of amino acids, carbohydrates, and lipids are already known and can be analyzed using automated procedures [7]. Moreover, in quantitative analysis of metabolites using LC/MS, substances which may only be present in small amounts within a biological sample can be precisely and quantitatively detected [8–11].

In forensic medicine, the analysis of endogenous metabolites also offers an innovative investigative approach, which has not yet been considered in the assessment of causes and circumstances of death. The investigation of the metabolome may result in the development of an additional diagnostic tool in the forensic workflow, but the systematic examination of body fluids collected postmortem may also supply additional clinical findings and allow to gain a deeper understanding of traumatological and pathophysiological processes.

In clinical diagnostics, e.g., for diagnosing neurological diseases, liquor cerebrospinalis (cerebrospinal fluid, CSF) is frequently used as a test substrate, as it communicates with the extracellular space of the central nervous system (CNS) without any barriers, and its analysis complements neuropathological investigations. For example, to assess primary and secondary brain damage following traumatic brain injury (TBI), numerous studies have been performed to find out whether the determination of the concentration profile of central nervous biomarkers in CSF might be suitable to diagnose (fatal) TBI and associated neuropathological changes in the brain parenchyma. Up to now, mostly structural proteins of the CNS cell compartments were analyzed in serum or CSF as markers for an acute trauma reaction [12–17].

Due to its protected anatomical location inside the skull and the spinal canal, CSF is a promising test substrate, which is described as relatively stable with only minor changes in the early postmortem phase and whose collection by suboccipital puncture or storage following removal of the brain can be easily integrated into the autopsy as standard procedure of the postmortem [18], thus supplying additional information for the neuropathological routine. In the medicolegal field, CSF was primarily used for biochemical investigations in routine work so far, for example, to determine the metabolic state or the postmortem interval [19–22]. In more recent studies, it was demonstrated that postmortem levels of cytokines, acute-phase proteins, and different CNS biomarkers [13, 23–25] in body fluids or sodium-glucose transporters [26] in brain tissue can also be used as an additional tool in forensic neuropathological diagnostics. Further, postmortem immunocytochemical stains of CSF are promising for determination of CNS involvement in the death progress [27].

The aim of this given method paper is to illustrate the possibilities of high-resolution LC/MS for the forensic examination of body fluids, especially CSF, in terms of metabolomics to make suggestions for the technical equipment and handling under routine and daily practice conditions and to analyze their potential as a new generation of biomarkers for diagnosing TBI and associated neuropathological changes in the CNS in the differentiation of other causes of death.

## Material and methods

### Material

The samples investigated included 10 cases of cardiovascular fatalities as a specific example of any sudden death originating from an inner cause (CVF; 3 females and 7 males aged between 42 and 90 years), 10 cases of isolated torso trauma without traumatic impact to the head (ITT; 3 females and 7 males aged between 53 and 86 years), 11 cases of acute deaths after TBI with survival time < 2 h (4 females and 7 males aged between 53 and 91 years), and 10 cases of multi-organ failure (MOF; 5 females and 5 males aged between 33 and 91 years), all autopsied at the Institute of Forensic Medicine of the University of Wuerzburg. ITT cases showed only very short survival phases in general, and all included CVF cases died suddenly or were found dead after a short period of time unseen. The survival time of TBI cases ranged between immediately in cases of opened TBI/pons dehiscence up to 1.5 h in cases of cortical contusions with final cardiopulmonary resuscitation. MOF cases were found dead in bed during hospitalization. The postmortem interval at autopsy of all cases ranged between 2 and 13 days (mean 5 days). Case characteristics are displayed in detail in Table 1. This research study has been approved by the ethics committee of the Medical Faculty of the University of Wuerzburg (local number 203/15).

**Table 1:** Detailed case characteristics of all fatalities included in this study: cardiovascular fatalities (CVF), isolated torso trauma (ITT), traumatic brain injury (TBI), and multi-organ failure (MOF)

Case number	Sex	Age	PMI	Cause of death
CVF				
1	M	50	2 days	Sudden cardiac death
2	M	81	5 days	Acute myocardial infarction
3	M	62	6 days	Pulmonary thromboembolism
4	F	87	3 days	Sudden cardiac death
5	M	82	6 days	Sudden cardiac death
6	F	53	5 days	Pulmonary thromboembolism
7	M	88	5 days	Acute myocardial infarction
8	F	42	5 days	Pulmonary thromboembolism
9	M	90	6 days	Sudden cardiac death
10	M	80	4 days	Acute myocardial infarction
ITT				
1	M	54	5 days	Pelvic trauma (car accident)
2	F	86	5 days	Pelvic trauma (car accident)
3	F	53	7 days	Thorax and pelvic trauma (bicycle accident)
4	M	65	5 days	Thorax trauma (bicycle accident)
5	M	63	4 days	Thorax trauma (industrial accident)
6	F	74	9 days	Pelvic trauma (car accident)
7	M	64	11 days	Pelvic trauma (car accident)
8	M	80	4 days	Pelvic trauma (fall)
9	M	88	3 days	Pelvic trauma (fall)
10	M	86	3 days	Pelvic trauma (fall)
TBI				
1	M	73	8 days	Subdural/subarachnoidal hemorrhage, opened TBI (car accident)
2	M	53	3 days	Subdural/subarachnoidal hemorrhage (car accident)
3	M	67	8 days	Cortical contusion, pons dehiscence (fall)
4	F	80	5 days	Cortical contusion (fall)
5	M	82	2 days	Cortical contusion (fall)
6	F	91	5 days	Cortical contusion (fall)
7	F	63	6 days	Cortical contusion (fall)
8	M	65	5 days	Cortical contusion (fall)
9	F	87	6 days	Cortical contusion (fall)
10	M	81	4 days	Cortical contusion (fall)
11	M	53	11 days	Subdural/subarachnoidal hemorrhage, pons dehiscence (car accident)
MOF				
1	F	61	7 days	Peritonitis/sepsis
2	M	91	6 days	Pericarditis/sepsis
3	M	80	3 days	Paralytic ileus/sepsis
4	F	84	10 days	Pericarditis/sepsis
5	M	70	3 days	Pneumonia/sepsis
6	F	76	5 days	Pneumonia/sepsis
7	F	91	4 days	Pneumonia/sepsis
8	M	68	13 days	Pneumonia/sepsis
9	M	33	5 days	Pneumonia/sepsis
10	F	74	9 days	Pneumonia/sepsis

### Preparation

Collection of CSF by suboccipital puncture was done using a disposable needle during dissection of the head and storage in polypropylene test vessels. Cases with obviously bloody/reddish CSF were excluded. Centrifugation of the sample should be performed directly after collection at 5000 rpm for 5 min at 4 °C. Until further laboratory processing, the CSF supernatant should be stored at − 80 °C.

### Extraction of metabolites using a modified B/D extraction

This step was done according to Bligh and Myer [28]. Twenty-five microliters of supernatant sample, 190 μl MeOH, 20 μl external standard (1 mM lamivudine in MeOH/CHCl_3_ (1/1; v/v))), and 30 μl 0.2 M HCl were mixed vigorously. After addition of 90 μl CHCl_3_, 100 μl CHCl_3_, and 100 μl H_2_O and vigorous mixing after each addition, the resulting mixture was centrifuged (2 min 20,000 rcf). Upper phase (UP) and lower phase (LP) were transferred to separate Eppendorf tubes and evaporated to dryness (UP by evaporation at 35 °C under a stream of N_2_ gas; LP in a vacuum concentrator).

For LC/MS analysis, the UP residues (containing water-soluble metabolites) were redissolved in 250 μl of 5 mM NH_4_OAc in CH_3_CN/H_2_O (25/75, v/v), and the LP residues (containing lipids) were redissolved in 50 μl 2-propanol.

### LC/MS analysis

The equipment used for LC/MS analysis was a Thermo Scientific Dionex Ultimate 3000 UHPLC system hyphenated with a Q Exactive Mass Spectrometer (QE-MS) equipped with a heated electrospray ionization (HESI) probe (Thermo Scientific, Bremen, Germany).

#### LC/MS analysis of water-soluble metabolites

Chromatographic separation of water-soluble metabolites of UP was achieved by applying 3 μl of dissolved sample on a SeQuant ZIC-HILIC Column (3.5-μm particles, 100 × 2.1 mm; Merck, Darmstadt, Germany), combined with a Javelin particle filter (Thermo Scientific) and a SeQuant ZIC-HILIC Precolumn (5-μm particles, 20 × 2 mm; Merck) using a linear gradient of mobile phase A (5 mM NH_4_OAc in CH_3_CN/H_2_O (5/95, v/v)) and mobile phase B (5 mM NH_4_OAc in CH_3_CN/H_2_O (95/5, v/v)). The LC gradient program was 100% solvent B for 2 min, followed by a linear decrease to 40% solvent B within 16 min, then maintaining 40% B for 6 min, then returning to 100% B in 1 min and 5 min 100% solvent B for column equilibration before each injection. The column temperature was set to 30 °C; the flow rate was maintained at 200 μl/min. The eluent was directed to the HESI source of the QE-MS from 1.85 to 20.0 min after sample injection.

#### LC/MS analysis of lipids

Chromatographic separation of lipids in LP was achieved by applying 3 μl of dissolved sample on an Acclaim RSLC 120 C8 (2.2-μm particles, 50 × 2.1 mm), combined with a Javelin particle filter and an Acclaim 120 C8 (5-μm particles, 10 × 2 mm; all Thermo Scientific) precolumn using a linear gradient of mobile phase A (CH_3_CN/H_2_O/formic acid (10/89.9/0.1, v/v/v)) and mobile phase B (CH_3_CN/2-propanol/H_2_O/formic acid (45/45/9.9/0.1, v/v/v/v)). The LC gradient program was 20% solvent B for 2 min, followed by a linear increase to 100% solvent B within 5 min, then maintaining 100% B for 27 min, then returning to 20% B in 1 min and 5 min 20% solvent B for column equilibration before each injection. The column temperature was set to 40 °C; the flow rate was maintained at 350 μl/min. The eluent was directed to the HESI source of the QE-MS from 2.0 to 29.0 min after sample injection.

### MS parameter settings

Scan type: full MS in alternating pos./neg. mode; resolution 70,000; AGC-target 3E6; maximum injection time 200 ms; and scan range 200–1500 *m*/*z*. HESI source parameters: sheath gas flow rate 30; auxiliary gas flow rate 10; sweep gas flow rate 3; spray voltage 2.5 kV in pos. mode and 3.6 kV in neg. mode; capillary temperature 320 °C; S-lens RF level 55.0; aux gas heater temperature 120 °C.

Peaks corresponding to the calculated lipid masses (MIM ± H^+^ ± 2 mMU) were integrated using TraceFinder software (Thermo Scientific). Specific peak areas were normalized to total lipid peak areas.

Ultrapure water was obtained from a Millipore water purification system (Milli-Q Merck Millipore, Darmstadt, Germany). LC/MS solvents, LC/MS NH_4_OAc, and standard compounds were purchased from Merck.

**Fig. 2:**
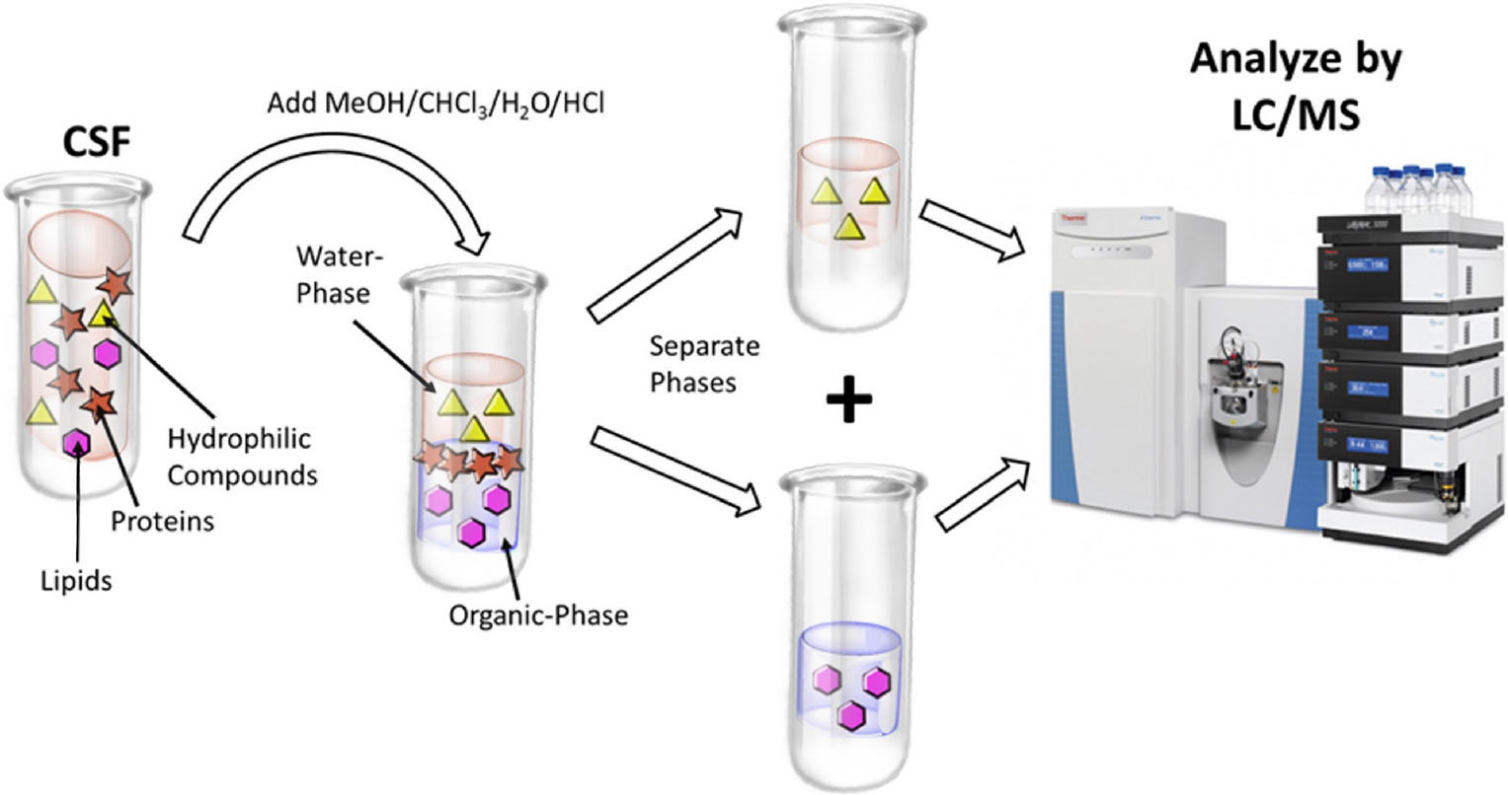
An illustration of the extraction of metabolites and LC/MS analysis. Flow diagram for the analysis of water-soluble metabolites and lipids in cerebrospinal fluid (CSF): first hydrophilic and lipophilic metabolites are extracted from CSF and separated from each other by a liquid/liquid phase distribution. The precipitated protein pellets have so far been stored but could be used for normalization or further proteomic analysis. Then, the components of the resulting fractions are separated using UHPLC and analyzed by means of high-resolution mass spectrometry

An illustration of the extraction steps of metabolites and LC/MS analysis is sketched in Fig. 2.

## Results and discussion

**Fig. 3:**
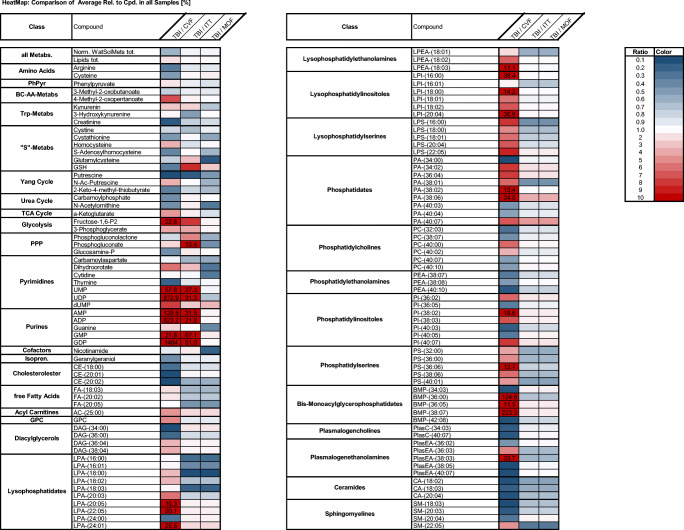
Heatmap of results. For the determination of the relative metabolite concentrations, the areas of the annotated MS signals were integrated (i) for the water-soluble metabolites normalized to the area of the lamivudine signal (as external standard) or (ii) for the lipids to the total of the areas of all annotated lipids. To compare the metabolite distribution within the different cohorts investigated (TBI to CVF, TBI to ITT, and TBI to MOF, respectively), quotients of the metabolite concentrations (i.e., metabolite A in CSF of individuals dying from TBI to the concentration of metabolite A in CSF of persons dying from CVF etc.) were depicted in the form of a heatmap where blue indicates a low value (comparable quotient between these two groups, ratio between 0 and 1) and red indicates a high value (relevant differences between the indices, ratios > 10)

In the metabolome analysis of postmortem CSF samples within the different cohorts investigated (TBI to CVF, TBI to ITT, and TBI to MOF, respectively), quotients of the metabolite concentrations show differences in nearly all of the here investigated 124 metabolites. In the metabolome investigation of TBI to CVF, the concentration ratios of bc-aa-metabolites (4-methyl-2-oxopentanoate), fructose-1,6-bisphosphate, UMP, UDP, AMP, ADP, GMP, GDP, lysophatidates (LPA-20:05, LPA-22:05, LPA-24:01), lysophosphatidylethanolamines (LPEA-18:03) lysophosphatidylinositols (LPI-16:00, LPI-18:00, LPI-20:04), phosphatidates (PA-38:02, PA-38:06), phosphatidylinositols (PI-38:02,), and bis-monoacylglycerophosphatidates (BMP-36:00, BMP-36:05, BMP-36:07) as well as plasmalogenethanolamines (PlasEA-38:03) were much higher than when compared TBI to ITT or MOF, respectively. By comparison of TBI to CVF, the concentration ratios of amino acids, creatinine, carbamoyl phosphate, thymine, geranylgeraniol, cholesterol esters, diacylglycerols, phosphatidylcholines (PC-32:03, PC-38:07, PC-40:07, PC-40:10), phosphatidylethanolamines, phosphatidylinositols (PI-36:05, PI-40:03, PI-40:05), bis-monoacylglycerophosphatidates (BMP-34:03, BMP-42:08), plasmalogencholines, plasmalogenethanolamines (PlasEA-36:02, PlasEA-38:05, PlasEA-40:07) ceramides, and sphingomyelins (SM-18:03, SM-20:03, SM-20:04) were lower than when comparing TBI to ITT or TBI to MOF, respectively. In the analysis of TBI to ITT the concentration ratios of GSH, phosphogluconate was higher as in the ratios for TBI to CVF or TBI to MOF. Interestingly, the same pyrimidines and purines as in the “TBI to CVF” comparison again showed high ratios. When comparing TBI to MOF, the concentration ratios of dihydroorotate, cytidine, guanine, and cofactors were lower than in both other groups compared. See heat map illustration of the results in Fig. 3.

According to the here given first experiences in the quantitative analysis of metabolites in CSF as a “model” of useful postmortem body fluids using LC/MS, it seems to be more appropriate to study substance groups/metabolite classes rather than analyzing individual molecules. We here used an empirically defined threshold of 10 in the absence of a gold standard, seeming robust enough to demonstrate relevant concentration ratios of different causes of death. However, the usefulness of a threshold like this remains to be answered in future studies.

CSF metabolomic profiling has been shown to be an important area with a promising number of applications in clinical medicine. Metabolomics is being increasingly studied for example in TBI, severe trauma, and shock conditions. The disruption of physiologic homeostasis in TBI was thought to be reflected in the metabolomics status [29, 30]. There may be nearly 2500 molecules that are affected by TBI and can be identified even far away from the CNS in urine [31]. We here demonstrated some of them to be relevantly elevated in cadaveric CSF of TBI deaths compared with other traumatic and natural causes of death.

Traditionally, autopsy and subsequent investigations such as histology, toxicology, and postmortem biochemistry allow us to determine the cause of death in about 90–95% of all cases [32, 33]. In a small percentage of cases, which varies depending on age, the cause of death is assumed to be a functional process that cannot be detected by the historical investigation methods or morphologically alone [34–37]. Lately, a “molecular autopsy” was able to demonstrate that genetic mutations may have a functional relevance, e.g., in cardiac arrhythmias, thus furnishing an explanation for part of these deaths [38]. However, not all functional events, which may be responsible for death, are based on such genetic mutations.

From a forensic point of view, stable test substrates, i.e., markers not subject to major postmortem changes, are promising for biochemical tests and methods [39], given the chance to reflect functional events after traumatization of tissue following TBI or hormonal fluctuations in metabolic deaths. Recent studies succeeded in using clinical biomarkers also in body fluids, especially CSF, which is less prone to autolysis compared with cadaveric blood or serum, with the objective to extend the diagnostic possibilities of forensic neuropathology [23, 25, 40, 41].

An innovative research approach not yet used in the forensic field widely is interpreting metabolomes as a new generation of biomarkers which can supply information on pathophysiological processes in the deceased and may be used as an additional diagnostic tool to determine the causes and circumstances of death. Due to the special feature of metabolites to rank at the end of the biochemical flow model after DNA and protein expression, metabolomics is implemented to identify the presence of abnormal levels of metabolites that are specific/a surrogate for an underlying disease process which is not primarily directed toward the causes of disease but rather shows the final results of metabolic functions including their alterations. As an example, increased plasma glucose levels do not represent the cause of diabetes mellitus but rather identify one of the endpoints of diabetes.

A major problem in forensic studies is to determine whether or not biochemical findings in autopsy tissues and body fluids are reasonably representative of those which prevailed during life. Agonal and postmortem factors like duration of agony and postmortem interval (PMI) as well as different medical characteristics of the deceased (i.e., age, gender, BMI and lifestyle, severity of disease, difference in medication) could introduce disease/death-unrelated changes which might affect the reliability of experimental data. For this reason and to minimize potential selection bias of typical confounders, the four groups of different causes of death were strictly selected age-matched (Kruskal-Wallis test *p* = 0.98), gender-matched (chi-square test *p* = 0.77), and PMI-matched (Kruskal-Wallis test *p* = 0.73).

Previous studies have shown alterations in expression levels of structural proteins and specific enzymes in the integrity of nucleic acids as well as changes in the morphology of neurons, receptors of neurotransmitters, or their transporters at different PMI [42–44]. In postmortem studies, caution is required especially in anatomical areas with high sensitivity for oxidative damage and conditions of anoxia. The hippocampus is especially sensitive to oxidative stress [45, 46]. In metabolome studies focused on the hippocampus of the mouse, the levels of many relevant metabolites increased until 2 h PMI and then their levels remained stable up to 5 h PMI [47]. However, 2 or 5 h of PMI is no realistic time frame before sampling or autopsy for forensic pathologists in most countries of the world.

Furthermore, since metabolomics is a promising technique for mapping the brain, multivariate analysis indicated significant differences between brain areas (cerebellum, brain stem, thalamus, frontal cortex, and ventral cortex). The brain regions with high interaction showed more similarity in metabolite signatures [6, 48]. If these imbalances are also represented in CSF of different locations is undetermined to date. Even considering this limitation, the identification of metabolites, which are stable or susceptible to change in combination with neuroanatomical characteristics, is challenging, but their investigation might help and shed light in interpretations of biochemical findings in autopsied tissue and body fluids.

According to our proof-of-concept evaluation, we assume that it is not the different concentrations between individual metabolites in CSF but the combination of several metabolites with different distributions that may provide information to the underlying biochemical processes and can thus be regarded as potential biomarker groups of different but common (final) entities of death.

Since metabolomics are nearly untouched yet in forensic pathology [49], we are not aware of “baseline postmortem data” for all the here presented metabolite classes which are accompanied by the death progress itself rather than differentiating fatal pathways. At this stage, we cannot discern whether altered levels of a particular metabolite or cocktails of metabolites are the direct consequence of the abnormal function of a particular pathway or an epiphenomenon which is linked to the cause of death. In our first metabolome investigation, we intentionally used heterogeneous, but confounder-matched study material with a wide range of PMI and age, which may influence the distribution of metabolites in postmortem CSF samples but represent the realistic quality of our daily autopsy material. For valid conclusions on potential and apparently stable metabolites, which may be useful as a “marker cocktail” to identify unclear causes of death with the help of a laboratory assessment by metabolomics, more detailed investigations are necessary with an increase of the postmortem CSF samples investigated and an intensive study of the metabolite constellation in different causes of death (natural and non-natural) also beyond the four investigated here (e.g., intoxication, suffocation, metabolic, and others) as well as the systematic investigation of pre mortem factors (age, gender, BMI, medication, and agonal state) and postmortem parameters (postmortem interval, conditions of storage). This higher caseload will also allow rigorous statistical evaluation of the results and determination of appropriate thresholds with calculation of statistically significant differences between the different groups. For this method paper, we intentionally decided to present the results of the analysis descriptively and with relative changes (ratios). Another aim would be to test to what extent other body fluids (e.g., femoral serum, vitreous humor, pericardial fluid) are also suitable to measure endogenous metabolic products and to use them for forensic diagnostic purposes.

Once basic methodological work has been completed, it is possible that, in the future, metabolome analysis in postmortem body fluids, especially CSF with its apparent stability against autolysis, may become an additional diagnostic tool in the interpretation of the circumstances of death (“liquid autopsy”) and can be regarded as a diagnostic additive to the forensic methodological spectrum of neuropathological processes (“neuroforensomics”) which can also reflect the continuous technical development of forensic medicine with an ongoing focus on the application of medical and scientific knowledge to solve specific issues of their own field.
